# Ferroptosis in Chronic Liver Diseases: Opportunities and Challenges

**DOI:** 10.3389/fmolb.2022.928321

**Published:** 2022-06-03

**Authors:** Xiaoxi Zhou, Yadong Fu, Wei Liu, Yongping Mu, Hua Zhang, Jiamei Chen, Ping Liu

**Affiliations:** ^1^ Key Laboratory of Liver and Kidney Diseases, Ministry of Education, Institute of Liver Diseases, Shuguang Hospital Affiliated to Shanghai University of Traditional Chinese Medicine, Shanghai, China; ^2^ Shanghai Key Laboratory of Traditional Chinese Clinical Medicine, Shanghai, China; ^3^ Institute of Interdisciplinary Medicine, Shanghai University of Traditional Chinese Medicine, Shanghai, China; ^4^ State Key Laboratory of Cell Biology, Center for Excellence in Molecular Cell Science, Shanghai Institute of Biochemistry and Cell Biology, Chinese Academy of Sciences, Shanghai, China

**Keywords:** ferroptosis, cell death, iron metabolism, lipid peroxidation, chronic liver diseases, liver fibrosis, hepatocellular carcinoma

## Abstract

Ferroptosis, an iron-dependent non-apoptotic cell death characterized by lipid peroxidation, is a cell death pathway discovered in recent years. Ferroptosis plays an important role in tumors, ischemia-reperfusion injury, neurological diseases, blood diseases, etc. Recent studies have shown the importance of ferroptosis in chronic liver disease. This article summarizes the pathological mechanisms of ferroptosis involved in System Xc−, iron metabolism, lipid metabolism, and some GPX4-independent pathways, and the latest research on ferroptosis in chronic liver diseases such as alcoholic liver disease, non-alcoholic fatty liver disease, liver fibrosis, hepatocellular carcinoma. In addition, the current bottleneck issues that restrict the research on ferroptosis are proposed to provide ideas and strategies for exploring new therapeutic targets for chronic liver diseases.

## Introduction

Ferroptosis is a type of regulatory cell death discovered in recent years. It is an iron-dependent non-apoptotic cell death characterized by lipid peroxidation (Dixon et al., 2012), and was first proposed by Dixon *et al.* in 2012 (Li J. et al., 2020). Ferroptosis is distinct from apoptosis, necrosis, and other well-characterized types of regulated cell death, and is characterized by rounded cell morphology, smaller mitochondria, and rupture of the outer mitochondrial layer, but no rupture of the plasma membrane, reduced cristae, nuclei of normal size, and no chromatin condensation (Dixon et al., 2012).

There are many types of ferroptosis inducers, which can be roughly divided into four categories. The first type includes erastin, sulfasalazine, glutamate, and p53, which target the cystine/glutamate antiporter, and are associated with impaired cystine uptake, glutathione (GSH) depletion, and loss of glutathione peroxidase 4 (GPX4) activity. The second type of ferroptosis inducers, including RSL3 and DP17, directly inhibit the activity of GPX4, and their mechanism is related to the covalent interaction with GPX4 and the inhibition of enzyme activity. The third type of ferroptosis inducers are those that induce ferroptosis by depleting GPX4 and CoQ10, such as FIN56. The last type of ferroptosis inducers, such as FINO2 (1,2-dioxane-containing endoperoxide), induce ferroptosis by promoting iron oxidation, driving lipid peroxidation and indirect inactivation of GPX4 (Feng and Stockwell, 2018; Capelletti et al., 2020). The inhibitors of ferroptosis are mainly divided into lipid autoxidation inhibitors and lipoxygenase inhibitors, according to the lipid peroxidation pathway. The former includes compounds that react with chain free radicals and can inhibit the autoxidative chain reaction, such as ferrostatin-1 and liproxstatin-1 (Xie et al., 2016). The latter can inactivate the enzymatic activity by removing the active site that binds iron (e.g., iron chelators) (Angeli et al., 2017).

The liver is one of the main storage sites of iron, and is also the main source of hepcidin, an important hormone regulating iron metabolism (Capelletti et al., 2020). Many chronic liver diseases share the characteristics of ferroptosis such as abnormal iron metabolism and lipid peroxidation, indicating that ferroptosis is closely related to chronic liver disease. Current researches on the role and mechanisms of ferroptosis in the pathogenesis and progression of chronic liver diseases are sorted out, and the advantages and limitations of the existing researches are discussed, which may provide new ideas for the treatment of chronic liver diseases.

## Pathological Mechanisms of Ferroptosis

**FIGURE 1:**
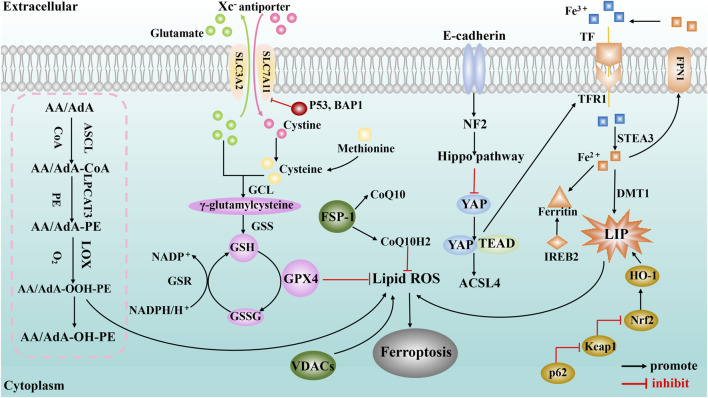
A schematic illustration showing the main mechanisms of ferroptosis. The purple spheres represent pathways centered on GPX4 (including the system Xc^−^ amino acid transport system, p53 regulation, BAP1 regulation and transsulfuration pathway); the pink dashed box represents the lipid metabolism pathway; the orange shapes represent signaling pathways centered on iron metabolism (including the golden ovals representing the p62-Keap1-Nrf2 regulatory pathway and IREB2); and the green spheres represent the regulation of ferroptosis by mitochondria (VDACs) and ferroxidase inhibitory protein 1-coenzyme Q10 (FSP-1-CoQ10).

The pathological mechanism of ferroptosis is mainly related to System Xc− malfunction, abnormal iron metabolism, abnormal lipid metabolism, etc. (Figure 1).

### System Xc^−^ Malfunction

System Xc− and GPX4 are the core regulatory targets in amino acid metabolism of ferroptosis. System Xc− is a sodium-independent antiporter composed of the heavy chain solute carrier family 3 member 2 (SLC3A2) and the light chain solute carrier family 7 member 11 (SLC7A11) (Lewerenz et al., 2013). System Xc− exchanges extracellular cystine and intracellular glutamate in a 1:1 ratio in an ATP-dependent manner; subsequently, the cystine transported into the cell is reduced to cysteine, which is used to synthesize the main antioxidant GSH in the cell (Dixon et al., 2012). Studies have shown (Wang L. et al., 2020) that inhibiting the activity of system Xc− can reduce the synthesis of intracellular GSH. The synthesis of GSH is essential to maintain the activity of GPX4, which is a physiological regulator of antioxidative damage (Capelletti et al., 2020). Once GPX4 is inactivated, it can cause severe lipid peroxidative damage, which, in turn, triggers ferroptosis (Ursini and Maiorino, 2020); hence, it is considered to be the main regulator of ferroptosis. Malfunctioning of system Xc− will affect the synthesis of intracellular GSH, limiting the superoxide-scavenging efficiency of GPX4, causing excessive lipid peroxidation and ferroptosis. The function of system Xc− is also affected by a variety of tumor suppressors. p53 is a tumor suppressor with extensive and powerful functions, and it has been reported to promote ferroptosis (Liu and Gu, 2022). Studies (Jiang et al., 2015) have shown that p53 (especially acetylation deficient mutant p53) can suppress cystine uptake by inhibiting the expression of SLC7A11, resulting in insufficient intracellular GSH synthesis, affecting the normal function of GPX4 and rendering cells sensitive to ferroptosis. Another study (Zhang Y. et al., 2018) suggests that the tumor suppressor BRCA1-associated protein 1 (BAP1) also promotes ferroptosis by downregulating the expression of SLC7A11. BAP1 encodes a nuclear deubiquitination enzyme to reduce histone 2A ubiquitination (H2Aub) on chromatin, and inhibits cystine uptake by inhibition of SLC7A11 expression, which leads to lipid peroxidation and ferroptosis.

### Abnormal Iron Metabolism

Iron homeostasis is very important for maintaining the functions of various tissues and organs in the body (Yu et al., 2020). In the human body, iron exists mainly in two forms, Fe^2+^ and Fe^3+^. After Fe^3+^ binds to transferrin in plasma, the iron-bearing transferrin binds to transferrin receptor 1 (TFR1) on cell membranes and enters cells by endocytosis. Then, Fe^3+^ is released from transferrin in an acidic environment and reduced to unstable and highly reactive Fe^2+^, a part of which enters the cytoplasmic labile iron pool (LIP), and another part of Fe^2+^ can be absorbed by ferritin or hemoglobin–protein complexation. In addition, Fe^2+^ can be excreted through ferroportin 1 (FPN1) *via* ferritin to maintain iron homeostasis inside and outside the cell.

Iron overload is an important cause of ferroptosis. For example, intake of iron ions can accelerate the occurrence of ferroptosis, which is a phenomenon that does not occur when supplementing other metal elements (Dixon et al., 2012); while increasing the expression of FPN1 promotes the extracellular release of iron ions, it can significantly reduce iron overload and reverse the occurrence of ferroptosis (Zhao et al., 2020). The Fenton reaction is an important source of reactive oxygen species (ROS) in the process of ferroptosis, which means that the reaction between Fe^2+^ and H_2_O_2_ can oxidize many known organic compounds such as carboxylic acids, alcohols, and esters to inorganic states and generate strong oxidative factors such as hydroxyl radicals (He et al., 2020). When the intracellular iron is overloaded, the excess Fe^2+^ exceeds the processing upper limit of the normal iron metabolism pathway and leads to the Fenton reaction, which can promote the production of lipid peroxidation and ROS, thereby triggering ferroptosis (Daher et al., 2017).

Other studies have shown that the p62-Keap1-Nrf2 signaling pathway may interfere with the occurrence and development of ferroptosis by affecting factors related to iron metabolism. Nrf2 is an important regulator of antioxidant responses, and studies have confirmed (Sun et al., 2016b) that under the intervention of ferroptosis inducers, the expression of p62 prevents the degradation of Nrf2 and increases the accumulation of Nrf2 protein by inactivating Keap1. Nrf2 protein further activates the expressions of retinoblastoma protein (Rb) and metallothionein-1G (MT-1G), and induces the transcriptional expressions of intracellular ferritin heavy chain (FTH1), NAD(P)H: quinone oxidoreductase 1 (NQO1), and heme oxygenase 1 (HO-1). The upregulation of HO-1 can promote the release of iron in heme and increase Fe^2+^ in LIP, thereby promoting the occurrence of lipid peroxidation and ferroptosis. Interestingly, in a recent study of 3D cancer spheroid models coupled with CRISPR-Cas9 screens (Takahashi et al., 2020), Nrf2 hyperactivation was shown to inhibit ferroptosis and promote the proliferation and survival of lung tumor spheroids. Targeting Nrf2 and GPX4 can kill tumor spheroids overall. This opens up a new perspective for studying the role of Nrf2 in the mechanism of ferroptosis, and also indicates the complexity of Nrf2 in ferroptosis. In addition, studies have reported that iron response element-binding protein 2 recombinant protein (IREB2) (Dixon et al., 2012) and heat shock 27 kDa protein 1 (Sun et al., 2016b) affect iron metabolism and downregulate the sensitivity of cells to ferroptosis by inhibiting the expression of TFR1.

### Abnormal Lipid Metabolism

Lipid peroxidation caused by abnormal lipid metabolism is one of the main mechanisms of ferroptosis, and lipid peroxidation of membrane phospholipids containing polyunsaturated fatty acid (PUFA) chains is the core of ferroptosis. Lipid peroxidation can oxidize PUFAs to peroxides, which affects the normal functions of cells. For example, it has been reported (Feng and Stockwell, 2018) that lipid peroxidation disrupts ion gradients and changes the fluidity and permeability of cell membranes.

However, the mechanism by which the lipid peroxidation of PUFAs affects cell ferroptosis warrants further exploration. As is known, overproduction of lipid peroxides can lead to their accumulation, triggering ferroptosis. The formation of lipid peroxides in PUFAs requires the participation of arachidonic acid lipid oxygenases (ALOXs), acyl-CoA synthase long-chain family 4 (ACSL4), and lysophosphatidylcholine acyltransferase 3 (LPCAT3) (Lei et al., 2019), and they are closely related to the occurrence of ferroptosis. Studies have found that ferroptosis in lung and intestinal tissue injury induced by ischemia-reperfusion is related to ACSL4 (Li et al., 2019; Xu et al., 2020). ACSL4 and LPCAT3 can regulate the sensitivity of cells to ferroptosis by affecting cellular lipid composition (Dixon et al., 2015; Doll et al., 2017). Silencing ALOXs can inhibit the occurrence and development of ferroptosis (Shintoku et al., 2017). Other pathways can also promote the synthesis of PUFAs, such as the decomposition of glutamine to glutamate, which is further converted into *α*-ketoglutarate, which also promotes the production of lipids, thereby increasing the formation of lipid peroxides and promoting the progression of ferroptosis.

Lipid peroxidation products of PUFAs, such as malondialdehyde (MDA) and 4-hydroxynonenal (4-HNE), induce ferroptosis through their cytotoxicity. PUFAs produce large amounts of MDA through chemical and enzymatic reactions (Tsikas, 2017) and are the most commonly measured biomarkers of lipid peroxidation. Moreover, the accumulation of 4-HNE and the formation of 4-HNE adducts caused by redox imbalance also aggravate the cytotoxic effect (Wang F. X. et al., 2020), which, in turn, increases the permeability of the cell membrane and causes ferroptosis.

### Other Mechanisms Associated With Ferroptosis

Although GPX4 is a core regulatory protein of ferroptosis, the responses of GPX4 inhibitors in different cancer cell lines are inconsistent, suggesting that other factors may regulate ferroptosis. Recent studies (Doll et al., 2019) have identified GPX4-independent pathways that also trigger ferroptosis, such as ferroptosis-suppressing protein 1 (FSP1), which was originally identified as apoptosis-inducing factor, mitochondrion-associated 2 (AIFM2). It protects against ferroptosis caused by GPX4 deletion, and the mechanism is related to CoQ 10 catalyzing the capture of lipid peroxyl radicals by NAD(P)H.

The E-cadherin-NF2-Hippo-YAP signaling axis is a non-cell-autonomous regulation of ferroptosis. Studies have found that epithelial cell ferroptosis is suppressed by E-cadherin-mediated intercellular interactions through activation of the intracellular NF2 (also known as merlin) and Hippo signaling pathway. Antagonizing NF2 enhances YAP activity to promote ferroptosis by upregulating several ferroptosis modulators, including ACSL4 and TFR1 (Wu et al., 2019).

Tumor suppressor p53 inhibits the expression of SLC7A11 and is involved not only in GPX4 -dependent ferroptosis but also in GPX4-independent ferroptosis by targeting ALOX12, iPLA2*β*, and SAT1/ALOX15 (Ou et al., 2016; Chu et al., 2019; Chen et al., 2021). It is worth noting that the role of p53 in ferroptosis is not only limited to the regulation of System Xc− function but also related to the regulation of lipid and iron metabolism (Liu and Gu, 2021). These results suggest that p53-mediated ferroptosis may be a basic and important ferroptosis pathway as GPX4-based ferroptosis. In addition, it has been reported (Friedmann Angeli et al., 2014; Feng and Stockwell, 2018) that the occurrence of ferroptosis is closely related to mitochondria, in that mitochondrial respiration can promote the production of ROS in cells, thereby promoting ferroptosis. The related mechanism has also been studied. For example (Li J. et al., 2020), the ferroptosis inducer erastin can directly act on the mitochondrial voltage-dependent anion channel (VDAC), which is not dependent on the GPX4 pathway.

## Role of Ferroptosis in Chronic Liver Diseases

**FIGURE 2:**
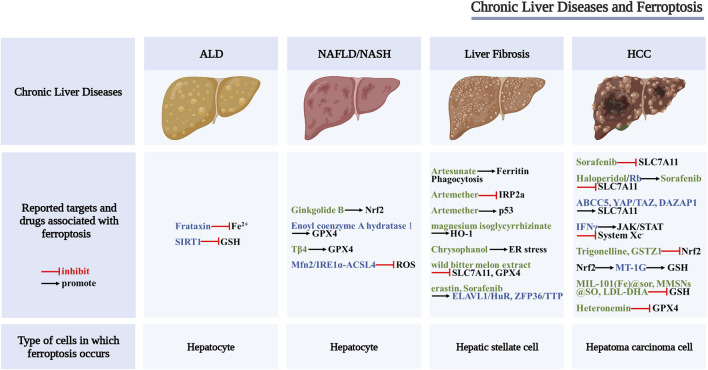
Scheme of the proposed role of ferroptosis in chronic liver diseases. Drugs and key regulators associated with ferroptosis (black font), potential regulators of ferroptosis (blue font), and potential therapeutic drugs for ferroptosis (green font).

Many studies have shown that ferroptosis characterized by System Xc− malfunction, abnormal iron metabolism, and abnormal lipid metabolism can be found in different chronic liver diseases, such as alcoholic liver disease, non-alcoholic fatty liver disease, liver fibrosis, and hepatocellular carcinoma, suggesting that targeting ferroptosis may provide new strategies for the treatment of chronic liver diseases (Figure 2).

### Ferroptosis and Alcoholic Liver Disease

Alcoholic liver disease (ALD), including alcoholic fatty liver disease, alcoholic hepatitis, alcoholic liver cirrhosis and their complications, is one of the most common types of chronic liver disease (Singal et al., 2018). A prospective follow-up study of patients with alcoholic cirrhosis found that hepatic iron overload exacerbates alcohol-induced liver damage and is associated with various adverse outcomes (Ganne-Carrié et al., 2000). Animal studies have shown that iron and alcohol can synergistically promote the development of ALD (Stål and Hultcrantz, 1993; Tsukamoto et al., 1995). Ferrostatin-1 can significantly reduce alcoholic liver injury *in vitro* and *in vivo* (Liu C.-Y. et al., 2020), suggesting that ferroptosis may play an important role in the progression of ALD.

Frataxin is a mitochondrial protein mainly involved in the body’s iron homeostasis and oxidative stress response. Frataxin deficiency can increase the iron content and lipid peroxidation level of LIP, mediate alcohol-driven ferroptosis, and promote the occurrence of ALD (Liu J. et al., 2020). Other studies have found that the deletion of intestinal SIRT1 can protect mice from alcohol-induced liver inflammation, and the mechanism is related to improving iron metabolism disorder, increasing liver GSH content, and alleviating lipid peroxidation damage. These results suggest that targeting intestinal SIRT1 to inhibit liver ferroptosis may have the potential of treating ALD (Zhou et al., 2020). In addition, clinical studies found decreased hepcidin levels in the liver of patients with ALD, suggesting that long-term alcohol consumption may cause iron overload in the liver, inducing ferroptosis (Costa-Matos et al., 2012).

### Ferroptosis and Nonalcoholic Fatty Liver Disease

Nonalcoholic fatty liver disease (NAFLD) is a liver disease characterized by the accumulation of lipid droplets, hepatocyte death, immune/inflammatory cell infiltration, and a certain degree of fibrosis. In some cases, NAFLD may develop into non-alcoholic steatohepatitis (NASH), which is a progressive liver disease that can further develop into liver fibrosis, liver cirrhosis, and hepatocellular carcinoma (Chalasani et al., 2018; Eslam et al., 2020). NAFLD alters iron metabolism in the body, and the progression of NAFLD is accompanied by extensive lipid accumulation. Owing to the close relationship between iron, lipid metabolism, and ferroptosis (Bozzini et al., 2005), it is of great significance to explore the relationship between ferroptosis and NAFLD.

Neither the specific inhibitor of programmed necrosis nor the knockout of the mixed lineage kinase domain-like protein MLKL could inhibit cell death, while the ferroptosis inhibitors trolox and DFO inhibited cell death in a methionine-/choline-deficient (MCD), ethionine-supplemented (CDE) diet-induced NASH mouse model, suggesting that the ferroptosis pathway is activated in the liver of NASH mice (Tsurusaki et al., 2019). Similarly, arsenic-induced NASH is also related to ferroptosis. The researchers found that the content of ACSL4, a key regulator of ferroptosis, was increased in the arsenic-induced rat NASH model. Further research found that inhibiting the Mfn2/IRE1α-ACSL4 pathway may be one of the important mechanisms to prevent the occurrence and development of NASH (Wei et al., 2020). It has been reported that the expression of GPX4 in mouse liver is decreased in the MCD combined with RSL3 mouse model, and treatment with GPX4 activator, iron chelator, or ferroptosis inhibitor can significantly slow the progression of NASH by enhancing GPX4 activity and affecting iron metabolism (Qi et al., 2020). In addition, the study by Li *et al.* showed that inhibition of ferroptosis could reduce the degree of liver fibrosis in a mouse NASH model induced by MCD, and the mechanism was related to reduction the level of lipid peroxidation to inhibit ferroptosis (Li X. et al., 2020). The above studies suggest that ferroptosis promotes the progression of NASH, and inhibition of ferroptosis may be a new approach to the treatment of NASH.

Currently, targeted ferroptosis therapy for NAFLD has also achieved some progress. Researchers found that T*β*4 can effectively improve liver lipid metabolism-related indicators in high-fat diet (HFD)-induced NAFLD rat models and inhibit the palmitic acid (PA)-induced cell death in hepatocyte line LO2. Combining T*β*4 with ferrostatin-1 can enhance the effect of T*β*4. However, either erastin or siRNA interference with GPX4 expression could attenuate the protective effect of T*β*4. This study suggests that T*β*4 may protect hepatocytes by inhibiting the GPX4 depletion-mediated ferroptosis pathway (Zhu et al., 2021). As a lipid metabolism enzyme, enoyl coenzyme A hydratase 1 (ECH1) is a key component of mitochondrial fatty acid *β*-oxidation. Studies have shown that ECH1 knockdown can aggravate liver steatosis, inflammation, and fibrosis in mice, while ferrostatin-1 intervention can alleviate the pathological changes aggravated by ECH1 knockdown, suggesting that ECH1 may slow the progression of NASH in mice by inhibiting ferroptosis (Liu B. et al., 2021).

In addition, studies have shown that traditional Chinese medicines or components of traditional Chinese medicines also play an important role in the treatment of NAFLD. Both HFD-induced ApoE^−/−^ mouse models and PA-/OA-induced HepG2 cells showed iron overload, upregulation of TFR1 expression, and downregulation of FTH1 expression. Ginkgolide B (GB) treatment can significantly increase the expression of Nrf2 and alleviate iron overload. These results suggest that GB may inhibit the progression of NAFLD by regulating the Nrf2 signaling pathway, inhibiting lipid accumulation and oxidative stress-induced ferroptosis in hepatocytes (Yang et al., 2020).

### Ferroptosis and Liver Fibrosis

Although liver fibrosis has been a global health concern for a long time, there is no FDA-approved effective drug to treat this condition, and the discovery of ferroptosis provides a new strategy to address this issue (Pan et al., 2021). Studies (Daher et al., 2017) have shown that the ferroptosis inhibitor ferrostatin-1 could effectively reverse liver fibrosis induced by a high-iron diet or CCl_4_ and ferroptosis induced by hepatic iron overload could aggravate acetaminophen-induced liver fibrosis in mice (Aldrovandi and Conrad, 2020), suggesting that hepatocyte ferroptosis promotes the progression of liver fibrosis. By contrast, when the target cells of ferroptosis are activated hepatic stellate cells (HSCs), the occurrence of ferroptosis can reduce liver fibrosis. SLC7A11 is a cystine/glutamate antiporter that can provide raw materials for intracellular synthesis of GSH (Dixon et al., 2012). Studies (Du et al., 2021) have shown that inhibiting the expression of SLC7A11 could induce ferroptosis in HSCs and alleviate liver fibrosis. Thus, ferroptosis acts as a “double-edged sword” in liver fibrosis. Given the important role of ferroptosis in the occurrence and development of liver fibrosis, the development of some drugs targeting ferroptosis has become a new research hotspot. For example, Zhang *et al.* (Zhang Z. et al., 2018; Zhang et al., 2020) found that erastin and sorafenib could induce ferroptosis in HSCs, thereby reducing liver fibrosis in mice. Their mechanism of action is related to the regulation of RNA-binding proteins such as ZFP36/TTP and ELAVL1/HuR. ZFP36/TTP inhibits ferroptosis, while ELAVL1/HuR promotes ferroptosis, which broadens the new molecular mechanism of ferroptosis to a certain extent.

There is growing evidence that natural products are safe and effective in preventing and treating liver fibrosis. Medications such as artesunate, artemether, magnesium isoglycyrrhizinate, chrysophanol, and wild bitter gourd extract have been confirmed to act on ferroptosis-related pathways and affect the progression of liver fibrosis. As reported by *in vitro* and *in vivo* studies (Kong et al., 2019), artesunate can alleviate liver fibrosis by triggering ferritin phagocytosis-mediated ferroptosis in HSCs. Artemether and magnesium isoglycyrrhizinate have also been shown to have good anti-fibrotic effects *in vitro* and *in vivo* (Sui et al., 2018; Li Y. et al., 2020), both of which can induce ferroptosis in HSCs. Artemether inhibits the ubiquitination of IRP2a and causes the accumulation of IRP2a in HSCs, thereby inducing the production of iron and ROS in HSCs and promoting the occurrence of ferroptosis. Other related studies (Wang et al., 2019) showed that the tumor suppressor gene p53 is an upstream molecule of artemether-induced ferroptosis in HSCs, suggesting that artemether can alleviate CCl_4_-induced hepatic fibrosis by promoting p53-dependent ferroptosis in HSCs. Heme oxygenase 1 (HO-1) is the upstream molecule of magnesium isoglycyrrhizinate-induced ferroptosis in HSCs, and siRNA knockdown of HO-1 can block magnesium isoglycyrrhizinate-induced ferroptosis in HSCs, thereby promoting liver fibrosis (Li Y. et al., 2020), suggesting that magnesium isoglycyrrhizinate alleviates CCl_4_-induced liver fibrosis by promoting HO-1-mediated ferroptosis in HSCs. Kuo et al. (2020) found that chrysophanol isolated from the rhizome of rhubarb can inhibit hepatitis B virus x protein (HBx)-induced activation of HSCs through ER stress and ferroptosis-dependent and GPX4-independent pathways, thereby reducing liver fibrosis. In addition, wild bitter melon extract can downregulate the protein levels of GPX4 and SLC7A11 in LPS-induced HSCs, suggesting that wild bitter melon extract can exert an anti-hepatic fibrosis effect by inducing ferroptosis (Ho et al., 2021). The exact effect of ferroptosis on chronic liver diseases is related to the cell type and the specific disease environment. In liver fibrosis, ferroptosis exerts opposite effects on hepatocytes and HSCs; the former aggravates liver damage, while the latter reduces liver damage. Therefore, ferroptosis may have completely opposite effects in different cell types, and the possible side effects can be largely alleviated if drug delivery systems targeting specific cell types can be developed.

### Ferroptosis and Hepatocellular Carcinoma

Hepatocellular carcinoma (HCC) is a leading cancer worldwide (Hartke et al., 2017). Sorafenib is an oral kinase inhibitor that inhibits tumor cell proliferation and angiogenesis, induces cancer cell apoptosis, and is a first-line molecular targeted drug for advanced HCC (Kong F.-H. et al., 2021). It is a ferroptosis inducer similar to erastin, which can induce ferroptosis in liver cancer cells, indicating that ferroptosis plays an important role in the killing effect of sorafenib on hepatoma carcinoma cells. The mechanism may be related to inhibiting the uptake of cystine by system Xc^−^, resulting in GSH depletion, loss of GPX4 activity, and accumulation of ROS (Galmiche et al., 2014). The study also found that the mechanism of sorafenib-induced ferroptosis in hepatoma carcinoma cells is related to Rb, which is a major negative regulator of cell proliferation and cell cycle progression. Rb-deficient hepatoma carcinoma cells are exposed to sorafenib. The cell death rate is much higher than that of hepatoma carcinoma cells with normal Rb protein level, and the mechanism may be related to the exposure of mitochondrial respiratory chain to sorafenib, which produces a large amount of ROS (Louandre et al., 2015).

Although sorafenib has a certain curative effect on the recurrence and metastasis of HCC, owing to the long-term exposure of patients to the drug, the sensitivity of hepatoma carcinoma cells to sorafenib gradually decreases. Therefore, the issue of drug resistance to sorafenib should be urgently addressed. The study of regulators that regulate SLC7A11, the core link of ferroptosis, will help to explore how to overcome the resistance to sorafenib in the treatment of HCC. For example, studies (Huang et al., 2021) have shown that ABCC5, as a novel regulator of ferroptosis in hepatoma carcinoma cells, can stabilize the SLC7A11 protein, increase the generation of intracellular GSH, and reduce the accumulation of lipid peroxidation products, thereby inhibiting ferroptosis, while downregulation of ABCC5 expression can significantly reduce the resistance of hepatoma carcinoma cells to sorafenib. Another study showed that the key driver of HCC resistance to sorafenib is YAP/TAZ, which not only induces the expression of SLC7A11 in a TEAD-dependent manner but can also maintain the stability, nuclear localization, and transcriptional activity of ATF4 protein, and then synergistically induce the expression of SLC7A11, thereby inhibiting sorafenib-induced ferroptosis (Gao et al., 2021) and increasing resistance to sorafenib. The chaperone molecule DAZAP1 of SLC7A11 mRNA plays an important role in HCC. DAZAP1 interacts with the 3ʹUTR (untranslated region) of SLC7A11 mRNA to regulate the stability of SLC7A11 (Wang et al., 2021b). In addition, activation of the p62-Keap1-Nrf2 pathway can resist sorafenib-induced ferroptosis in hepatoma carcinoma cells (Sun et al., 2016a). MT-1G is one of the target genes of the Nrf2 pathway. Clinical studies (Houessinon et al., 2016) have shown that the serum MT-1G protein level increased in patients with HCC treated with sorafenib, and that the deletion of MT-1G gene accelerated GSH depletion and lipid peroxidation. This indicates that MT-1G may promote sorafenib resistance in hepatoma carcinoma cells by inhibiting ferroptosis, suggesting that it can be used as a potential prognostic indicator for patients with advanced HCC receiving sorafenib. Some researchers also found that trigonelline, the main active ingredient of the traditional Chinese medicine fenugreek, and glutathione transferase zeta 1 (GSTZ1) can promote ferroptosis by acting on Nrf2, thereby reducing sorafenib resistance (Wang et al., 2021a). Some scholars have confirmed, for the first time, that exposure of hepatoma carcinoma cells to sorafenib can cause Nrf2 inactivation and passive upregulation of sigma receptor expression. Sigma receptor antagonist haloperidol can synergize with sorafenib to promote ferroptosis in hepatoma carcinoma cells (Bai et al., 2017; Bai et al., 2019).

In addition to the research on sorafenib, the development of drugs targeting ferroptosis-related pathways for the treatment of HCC has also become one of the research hotspots. Chang et al. (2021) discovered a marine terpenoid, heteronemin, which can reduce the expression of GPX4 and induce ferroptosis in hepatoma carcinoma cells, suggesting that heteronemin can be used as a potential drug for the treatment of HCC. IFN*γ* has also been confirmed to inhibit system Xc− activity by activating JAK/STAT signaling and increase the sensitivity of hepatoma carcinoma cells to ferroptosis, which will provide a new mechanism for the use of IFNγ in treating HCC (Kong R. et al., 2021). In addition, researchers have used nanotechnology to develop some HCC therapeutics based on the ferroptosis mechanism, such as sorafenib (sor)-loaded MIL-101(Fe) nanoparticles (NPs) [MIL-101(Fe)@sor] (Liu X. et al., 2021), MMSNs@SO (Tang et al., 2019) and LDL nanoparticles reconstituted with the natural omega-3 fatty acid docosahexaenoic acid (LDL-DHA) (Ou et al., 2017). These substances can deplete GSH and reduce the level of GPX4, and induce ferroptosis in hepatoma carcinoma cells. In conclusion, ferroptosis could become a potential target for the treatment of HCC.

## Limitations in Current Researches of Ferroptosis in Chronic Liver Diseases

The current research on ferroptosis has some limitations. Firstly, the research results are mostly based on animal models and lack reliable clinical evidence, and most researches are limited to the observation of curative effect, owing to a lack of a mature ferroptosis model and systematic in-depth mechanism research. Despite the existence of various types of ferroptosis inducers and inhibitors, they are rarely used in clinical practice. Secondly, many chronic liver diseases may have multiple cell death modes at the same time, and further research is needed on the connection between ferroptosis and other cell deaths. When regulating a specific cell death pathway, the influence of other cell death pathways must be taken into account. Moreover, the current researches on ferroptosis mostly focus on HCC, and research on other liver diseases is relatively lacking. In addition to the types of liver diseases mentioned in this article, it has also been reported (Kain et al., 2020) that Nutlin-3 (a commonly used, highly selective, membrane-permeable MDM2 antagonist) can reduce the plasmodium liver stage infection by activating the p53-SLC7A11-GPX4 signaling pathway. This indicates that the ferroptosis pathway is an effective immune barrier against parasites. The role of ferroptosis in many liver diseases is still largely unclear, and further in-depth research is warranted to reap more fruitful achievements in this field.

The most urgent issue to be addressed at present is the lack of specific and sensitive biomarkers and detection methods to clarify the characterization of ferroptosis. Currently, the occurrence of ferroptosis is usually judged by direct observation of cell morphological changes using transmission electron microscopy. Pathological tissue staining has been used to evaluate the accumulation of lipid peroxides, iron overload, etc., and the occurrence of ferroptosis was indirectly and comprehensively judged from different aspects by measuring lipid peroxidation and iron metabolism indicators related to ferroptosis.

## Conclusion

Ferroptosis, as a new form of cell death, is involved in the occurrence and development of various chronic liver diseases through a complex mechanism involving multiple pathways and links, which provides a new direction for the treatment of chronic liver diseases. In particular, it provides important ideas for combination therapy for HCC and the development of new drugs. The causes of cell ferroptosis include dysfunction of amino acid transporters, changes in iron homeostasis, and accumulation of lipid peroxidation. Many key proteins in the ferroptosis-related signaling pathway, such as GPX4, which regulate ferroptosis, may become drug targets. GPX4 is a key enzyme that has antioxidant properties and can catalyze the reduction reaction of lipid peroxides, thereby inhibiting the occurrence of ferroptosis. Previous studies on the mechanism of ferroptosis mostly relied on related mechanisms centered on GPX4. However, with the in-depth study of ferroptosis, more and more other ferroptosis regulations have been discovered. Surprisingly, p53 has been found to modulate ferroptosis by both the GPX4-dependent and GPX4-independent ferroptosis pathways, which suggests that targeting p53-mediated ferroptosis could be a potential treatment of chronic liver diseases, particularly HCC. In addition, recent studies have found that GPX4-independent mechanisms such as the FSP1 suppressor and the Hippo signaling pathway are also involved in the regulation of ferroptosis.

Existing research suggests that traditional Chinese medicine has a certain regulatory effect on ferroptosis. The occurrence of ferroptosis is inseparable from lipid accumulation and oxidative stress. There are many antioxidant components in traditional Chinese medicine, such as Schisandra chinensis and Salvia miltiorrhiza.

Targeting ferroptosis can be regarded as a new research idea for the treatment of chronic liver diseases. As a double-edged sword, inhibition of ferroptosis can be used to prevent and protect various chronic liver injuries caused by iron deposition and abnormal lipid metabolism, while induction of ferroptosis is helpful for the treatment of hepatocellular carcinoma. Further detailed studies on ferroptosis will provide important strategies for exploring new therapeutic targets and promoting new drug development for chronic liver diseases.
